# Supplementary material for: Initial aortic repair versus medical therapy for early uncomplicated type B dissections
Source: PLoS One. 2025 Mar 20;20(3):e0319561. doi: 10.1371/journal.pone.0319561 (PMC11957770; doi:10.1371/journal.pone.0319561)
Supplement: S1. Table — (DOCX) [file pone.0319561.s001.docx]

**Table S1 High risk features by initial management in uncomplicated type B aortic syndrome**

|  | **Aggressive (N=77)** | **Conservative (N=33)** | **P value** |
| --- | --- | --- | --- |
| Bloody pleural effusion | 2 (2.6%) | 0 | 1 |
| Aortic diameter≧40mm^a^ | 19 (24.7%) | 6 (18.2%) | 0.620 |
| Radiographic malperfusion | 9 (11.7%) | 0 | 0.055 |
| False lumen diameter≧22mm^a^ | 15 (35.7%) | 4 (26.7%) | 0.751 |
| Hematoma thickness≧8mm^b^ | 17 (48.6%) | 5 (27.8%) | 0.239 |

^a^ only counted in typical dissection; ^b^ only counted in atypical dissection
