# Supplementary material for: Initial aortic repair versus medical therapy for early uncomplicated type B dissections
Source: PLoS One. 2025 Mar 20;20(3):e0319561. doi: 10.1371/journal.pone.0319561 (PMC11957770; doi:10.1371/journal.pone.0319561)
Supplement: S2 Table — (DOCX) [file pone.0319561.s002.docx]

**Table S2 Procedural characteristics by initial management in uncomplicated type B aortic syndrome**

|  | **Aggressive (N=77)** | **Conservative (N=7)** |
| --- | --- | --- |
| Days from onset to stent-grafting, median (IQR) | 15 (8.5‒ 24) | 298 (138‒ 423) |
| Procedure detail |  |  |
| Stent graft per patient | 2 (1– 2) | 2 (1– 2) |
| Carotid-subclavian bypass | 30 (39.0%) | 5 (71.4%) |
| Carotid-carotid bypass | 1 (1.3%) | 0 |
| Carotid-carotid-subclavian bypass | 1 (1.3%) | 0 |
| Left subclavian artery embolization | 15 (19.5%) | 4 (57.1%) |
| Left subclavian artery stent or stent graft | 13 (16.9%) | 2 (28.6%) |
| Left common carotid artery stent or stent graft | 7 (9.1%) | 1 (14.3%) |
| Superior mesentery artery stent | 2 (2.6%) | 0 |
| Iliac artery stent | 3 (3.9%) | 0 |
| Total arch replacement under CPB | 5 (6.5%) | 0 |
| Arch debranching | 2 (2.6%) | 0 |
| Aortic bare stent (One/two) | 5 (6.5%) / 8 (10.4%) | 0 |
| Sternotomy | 7 (9.1%) | 0 |
| Proximal landing zone of stent graft |  |  |
| Dacron prosthesis | 5 (6.5%) | 0 |
| Zone 0 | 2 (2.6%) | 0 |
| Zone 1 | 8 (10.4%) | 1 (14.3%) |
| Zone 2 | 49 (64.6%) | 4 (57.1%) |
| Zone 3 | 10 (13%) | 0 |
| Zone 4 | 2 (2.6%) | 2 (28.6%) |
| Zone 5 | 1 (1.3%) | 0 |
| Brand of stent graft |  |  |
| Zenith (COOK) / TAG (Gore) / Valiant (Medtronic) | 35 / 28 / 14 | 3 / 2 / 2 |
| Pre/Post stent-grafting cerebrospinal fluid drainage | 0 / 1 (1.3%) | 0 |

CPB: Cardiopulmonary bypass
