# Supplementary material for: Initial aortic repair versus medical therapy for early uncomplicated type B dissections
Source: PLoS One. 2025 Mar 20;20(3):e0319561. doi: 10.1371/journal.pone.0319561 (PMC11957770; doi:10.1371/journal.pone.0319561)
Supplement: S3 Table — (DOCX) [file pone.0319561.s003.docx]

**Table S3 Outcomes by initial management in uncomplicated typical type B aortic dissection**

|  | **Aggressive (N=42)** | **Conservative (N=15)** | **P value** |
| --- | --- | --- | --- |
| **Early outcomes (≦30 days)** |  |  |  |
| Mortality | 1 (2.4%) | 0 | 1 |
| Aortic rupture | 1 (2.4%) | 0 | 1 |
| Acute myocardial infarction | 0 | 0 |  |
| Neurological event | 1 (2.4%) | 0 | 1 |
| Cerebral ischemia | 1 (2.4%) | 0 | 1 |
| Spinal cord | 0 | 0 |  |
| Major complications | 1 (2.4%) | 0 | 1 |
| Retrograde type A dissection | 1 (2.4%) | 0 | 1 |
| Acute kidney injury^a^ | 9 (21 %) | 0 | 0.05 |
| Post stent-grafting ischemic limb | 0 | X |  |
| Post stent-grafting GI bleeding | 0 | X |  |
| Post stent-grafting pneumonia | 2 (4.8%) | X |  |
| Post stent-grafting respiratory failure | 0 | X |  |
| Endoleak | 7 (16.7%) | X |  |
| Type 1b | 1 (2.4%) |  |  |
| Type 2 | 5 (11.9%) |  |  |
| Type 3 | 1 (2.4%) |  |  |
| Re-intervention | 0 | X |  |
| **Cumulative midterm outcomes** |  |  |  |
| All-Cause Mortality | 10 (23.8%) | 5 (33.3%) | 0.507 |
| Aorta-related Mortality | 1 (2.4%) | 0 | 1 |
| Retrograde Type A dissection | 1 () | 0 | 1 |
| Endoleak | 11 (26.2%) | 0 | 0.026 |
| Type 1 | 3 (7.1%) | 0 |  |
| Type 2 | 7 (16.7%) | 0 |  |
| Type 3 | 2 (4.8%) | 0 |  |
| Re-intervention | 9 (21.4%) | 2 (13.3%) | 0.71 |

Major complication: Cerebral ischemia, spinal cord ischemia, myocardial infarction and aortic rupture

^a^ increase in serum creatinine of≧0.5 mg/dL or increase to≧150% from baseline.
