# Supplementary material for: Initial aortic repair versus medical therapy for early uncomplicated type B dissections
Source: PLoS One. 2025 Mar 20;20(3):e0319561. doi: 10.1371/journal.pone.0319561 (PMC11957770; doi:10.1371/journal.pone.0319561)
Supplement: S4 Table — (DOCX) [file pone.0319561.s004.docx]

**Table S4 Outcomes by initial management in uncomplicated atypical type B aortic dissection**

|  | **Aggressive (N=35)** | **Conservative (N=18)** | **P value** |
| --- | --- | --- | --- |
| **Early outcomes (≦30 days)** |  |  |  |
| Mortality | 0 | 0 |  |
| Aortic rupture | 0 | 0 |  |
| Acute myocardial infarction | 0 | 0 |  |
| Neurological event | 5 (14.3%) | 0 | 0.153 |
| Cerebral ischemia | 3 (8.6%) | 0 | 0.543 |
| Spinal cord | 2 (5.7%) | 0 | 0.543 |
| Major complications | 5 (14.3%) | 0 | 0.153 |
| Retrograde type A dissection | 0 | 0 |  |
| Acute kidney injury^a^ | 4 (11.4%) | 0 | 0.287 |
| Post stent-grafting ischemic limb | 1 (2.9%) | X |  |
| Post stent-grafting GI bleeding | 1 (2.9%) | X |  |
| Post stent-grafting pneumonia | 2 (5.7%) | X |  |
| Post stent-grafting respiratory failure | 2 (5.7%) | X |  |
| Endoleak | 2 (5.7%) | X |  |
| Type 1b | 0 |  |  |
| Type 2 | 2 (5.7%) |  |  |
| Type 3 | 0 |  |  |
| Re-intervention | 2 (5.7%) | X |  |
| **Cumulative midterm outcomes** |  |  |  |
| All-Cause Mortality | 13 (37.1%) | 7 (38.9%) | 1 |
| Aorta-related Mortality | 0 | 1(5.6%) | 0.34 |
| Retrograde Type A dissection | 1 (2.9%) | 0 | 1 |
| Endoleak | 4 (11.4%) | 1(5.6%) | 1 |
| Type 1 | 0 | 0 |  |
| Type 2 | 4 (11.4%) | 0 |  |
| Type 3 | 0 | 1(5.6%) |  |
| Re-intervention | 8 (22.9%) | 0 | 0.04 |

Major complication: Cerebral ischemia, spinal cord ischemia, myocardial infarction and aortic rupture

^a^ increase in serum creatinine of≧0.5 mg/dL or increase to≧150% from baseline.
