# Supplementary figures and images for: Initial aortic repair versus medical therapy for early uncomplicated type B dissections
Source: PLoS One. 2025 Mar 20;20(3):e0319561. doi: 10.1371/journal.pone.0319561 (PMC11957770; doi:10.1371/journal.pone.0319561)

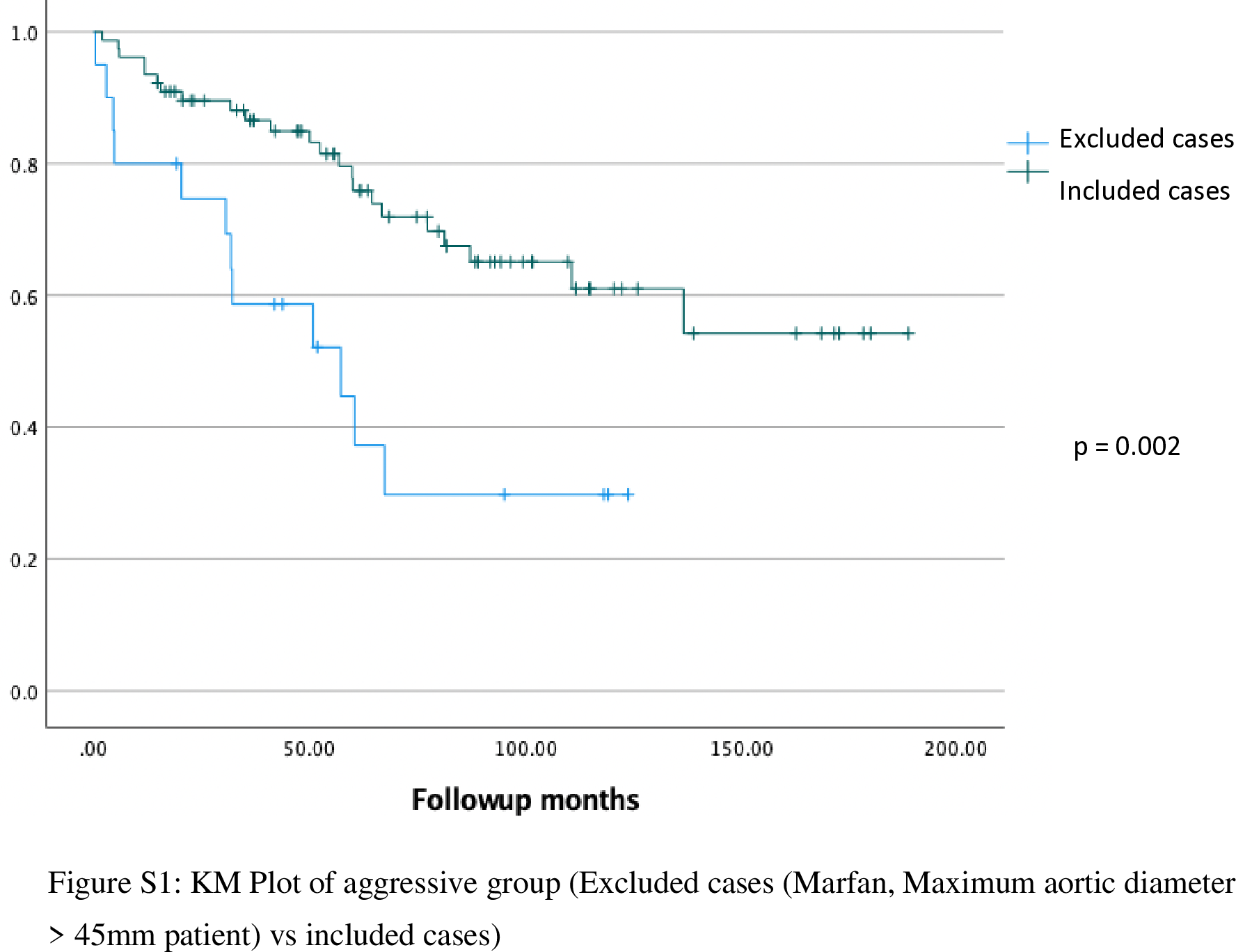

Supplement: S1 Fig — (TIF) [file pone.0319561.s005.tif]
