# Supplementary material for: Initial aortic repair versus medical therapy for early uncomplicated type B dissections
Source: PLoS One. 2025 Mar 20;20(3):e0319561. doi: 10.1371/journal.pone.0319561 (PMC11957770; doi:10.1371/journal.pone.0319561)
Supplement: Attachment — Submitted filename: Responses to Reviewers.docx [file pone.0319561.s009.docx]

PLOS ONE, EDITORIAL OFFICE

27 Oct 2024

Dear Reviewers or Editors,

The feedback provided by reviewers has been helpful to improving this manuscript and we are grateful for their input. We have also responded in detail to all the comments made in the following pages. This rebuttal follows the format in which the points from the reviewers were left in the original order, in blue, and our responses (in black) were then inserted after each of those points.

**Reviewer #1**

1. I would recommend shrinking the Introduction section, and moving the information from it to the Discussion section.

Authors: According to the reviewer’s suggestion, we have re-written the introduction and discussion sections.

2. The purpose of the study has not been defined.

Authors: We have added in last paragraph of Introduction. (Page 6, Line 102-104)

3. No data about amount of patients with retrograde aortic dissection is provided.

Authors: In Figure 1, 76 patients were excluded because of zone 0 involvement, and they were also known as retrograde aortic dissection cases (in the new reporting standards as B_0,x_).^1^

4. A significantly higher proportion of patients with signs of unfavourable prognosis in the interventional treatment group is noteworthy. This may have influenced the results obtained. To offset this effect, I would recommend averaging the patients according to the presence or absence of signs of unfavourable prognosis, all together or individually (maximum aortic diameter 22 mm or more, pleural effusion, refractory hypertension, etc.).

Authors: We would discuss how to manage this issue in answer to comment 9.

5. I would also recommend excluding patients with Marfan syndrome from the analysis, as there are few of them (3 in each group) and removing them would allow for more "clean" groups and more reliable results.

Authors: Marfan syndrome patients were excluded in revised version.

6. In addition, there is a question of whether transcatheter interventions in patients with Marfan syndrome and acute aortic syndrome are consistent with the recommendation to prefer open surgery in these patients.

Authors: Yes, transcatheter intervention was not recommended in Marfan patients. However, this is a retrospective study and some patients without typical characteristics were diagnosed with Marfan syndrome after operation by genetic test. It is not an issue in the revised manuscript as Marfan patients were excluded.

7. The question arises as to why the median time from the onset of dissection to surgery in the intervention group was 16 days (from 9 to 26 days) and how correct it is in this situation to talk about treating acute dissection.

Authors: We use “early” in the topic of this manuscript, which represented “acute and subacute” dissection. These patients are uncomplicated dissection cases and TEVAR could be done non-urgently, and beyond the acute phase. Furthermore, many studies suggested that performing TEVAR in subacute stage would be better. ^2-4^

8. I would like to know what were the indications for total arch prosthetics under CPB and if the frozen or conventional elephant trunk technique was used.

Authors: This is a retrospective study, and the indication of total arch replacement is not clearly mentioned in medical records. The decision to perform total arch replacement depends on surgeon in-charge, which usually associated with unhealthy aortic arch, arch anatomy or larger aortic arch diameter. Frozen elephant trunk was used in all total arch replacement cases of this study.

9. The conclusion of the worst results of aggressive treatment with early TEVAR is based on the incorrect comparison of two different groups. I would recommend performing “cleaning” and averaging of the study groups as mentioned above. To maximize the validity of the comparison, I would recommend that the authors discuss the possibility of performing a propensity score matching. The absence of differences between the results of different types of treatment indicates a correct selection of patients for medical, interventional and open surgical treatment, and the authors' task is to statistically process the data in such a way as to offset the effects of heterogeneous composition of the groups.

Authors: We tried to offset the effects of heterogeneous composition between these two groups. The association of survival and high-risk features were analyzed, and only “maximal aortic diameter” was strongly related to survival, and the others (ulceration thickness, radiographic malperfusion and IMH with ulcer-like projection) were not. Then distribution of the variable (maximal aortic diameter) was checked in two groups and found that more patients in the aggressive group had larger aortic diameters. Directly excluding patients whose maximal aortic diameter larger than 45mm can correct the pre-treatment difference of variables between the two groups without excluding too many cases (as performing Propensity Score Matching). Hence, we excluded patients with “maximal aortic diameter” larger than 45 mm in the revised manuscript.

10. I recommend performing English proofreading and major revision.

Authors: English proofreading was done.

Reviewer #2:

1. To be distribution-free and no need to account for normal distribution, please use non-parametric statistical methods for all analyses (your sample sizes were also not large), i.e. median+-IQR, Mann-Whitney U test (not t-test), and Fisher's exact test.

Answer: Statistical methods were corrected in the revised manuscript.
2. The presented tables are too complicated and please consolidate, for example, Tables 6 and 7. There were too many measurements. Possible to simplify them and just show aortic remodeling % or Yes/No?

Answer: Most tables and measurements have been simplified. However, details of aortic remodeling were kept as it demonstrated effect of TEVAR over different segment of aorta.
3. Plos ONE is a general-purpose journal, not a cardiovascular or aortic surgery journal. Please rewrite the whole manuscript to make it more reader-friendly to non-specialist audience. For example, please define "aortic remodeling" and explain its relationships to all your measurements in Tables 6 and 7.

Answer: Definition and evaluation of aortic remodeling was mentioned in the method section.

4. Please define "survival" or "mortality". Hospital survival? Overall survival? 30-day survival? Hospital mortality? All-cause mortality? Aorta-related mortality? Surgical mortality? 30-day mortality? Please define your hard and soft outcomes. Short-term? Long-term? How to define short- and long-term?

Answer: We have simplified measurements to 30-day, overall and aortic-related mortality. Definitions were mentioned in Method section. Outcomes were also simplified to short term (30 day) and mid-term (10 years), with major aorta related complications such as aorta rupture and aorta related mortality, procedural complications, neurological complications and else.

5. You said aggressive group had no survival advantage. Was it long-term, short-term, or overall? Then you said aortic remodeling in the aggressive group may translate into improved long-term survival. How and why? Were they contradicted viewpoints? Please explain with your data by a reader-friendly way.

Answer: In the revised manuscript, we focused on patients whose maximum aortic diameter ≤ 45mm. However, in this specific group, aortic remodeling in the aggressive group could not translate into improved mid-term survival.

6. An analog to your topic is "Initial CABG versus PTCA for early uncomplicated CAD-1VD". How do you think the data will show? CABG may show better long-term graft patency but was it worthy the surgical risks and would the patient/family accept it? Over-treatment may show superior efficacy but how about the risk, cost, or life-quality effect? Please discuss this kind of arguments in your discussion.

Answer: A short paragraph mentioned about cost-effectiveness was added in the discussion section of revised manuscript.

Reference:

1.Lombardi JV, Hughes GC, Appoo JJ, Bavaria JE, Beck AW, Cambria RP, Charlton-Ouw K, Eslami MH, Kim KM, Leshnower BG, Maldonado T, Reece TB and Wang GJ. Society for Vascular Surgery (SVS) and Society of Thoracic Surgeons (STS) Reporting Standards for Type B Aortic Dissections. *The Annals of thoracic surgery*. 2020;109:959-981.

2.Potter HA, Ding L, Han SM, Weaver FA, Beck AW, Malas MB and Magee GA. Impact of high-risk features and timing of repair for acute type B aortic dissections. *Journal of vascular surgery*. 2022;76:364-371.e3.

3.Jubouri M, Al-Tawil M, Yip HCA, Bashir A, Tan S, Bashir M, Anderson R, Bailey D, Nienaber CA, Coselli JS and Williams I. Mid- and long-term outcomes of thoracic endovascular aortic repair in acute and subacute uncomplicated type B aortic dissection. *Journal of cardiac surgery*. 2022;37:1328-1339.

4.Xie E, Yang F, Liu Y, Xue L, Fan R, Xie N, Chen L, Liu J and Luo J. Timing and Outcome of Endovascular Repair for Uncomplicated Type B Aortic Dissection. *European journal of vascular and endovascular surgery : the official journal of the European Society for Vascular Surgery*. 2021;61:788-797.
