# Supplementary material for: Initial aortic repair versus medical therapy for early uncomplicated type B dissections
Source: PLoS One. 2025 Mar 20;20(3):e0319561. doi: 10.1371/journal.pone.0319561 (PMC11957770; doi:10.1371/journal.pone.0319561)
Supplement: Attachment — Submitted filename: PONE-D-23-19689 _review 04 Jan 2025.docx [file pone.0319561.s010.docx]

An interesting paper by Jyh Shinn Teh and associates. The study is devoted to the relevant problem of early descending aortic repair or medical therapy in patients with early uncomplicated type B dissections. Some studies showed possibility of unpredictable progression of disease, which suggested the need for earlier intervention. The authors analyzed mortality and aortic remodeling in patients who underwent aggressive and conservative therapy. Importantly for further analysis, patients with Marfan syndrome, traumatic, iatrogenic, zone 0 (ascending aorta) involvement, and maximal aortic diameter >45mm were excluded.

The list of references used needs to be updated. Of the 32 papers used, 24 (75%) were published earlier than 2019 (more than 5 years ago), including 5 papers published in 2004 and earlier (20 or more years ago).

The text contains a number of typographical errors (e.g., on line 82) as well as some syntylic errors. I recommend performing English proofreading by a native speaker.

In general, the paper is technically sound, and the methods are appropriate and properly conducted. The results and conclusions logically follow from the conducted analysis. The data are adequately interpreted. The statistical analysis is sound. The claims are fully supported by the study data. There are no special ethical concerns regarding the use of human subjects. I have no other special comments on the work, and I recommend it for publication after minor revision.

Thank you for submitting your study to the Journal and good luck for the paper.

Vladimir Uspenskiy, MD, DrHabil, Associate Professor
