# Supplementary material for: Initial aortic repair versus medical therapy for early uncomplicated type B dissections
Source: PLoS One. 2025 Mar 20;20(3):e0319561. doi: 10.1371/journal.pone.0319561 (PMC11957770; doi:10.1371/journal.pone.0319561)
Supplement: Attachment — Submitted filename: Response to reviewers 0115.docx [file pone.0319561.s012.docx]

PLOS ONE, EDITORIAL OFFICE

15 Jan 2025

Dear Reviewers or Editors,

The feedback provided by reviewers has been helpful to improving this manuscript and we are grateful for their input. We have responded in detail to all the comments by reviewer #3 made in the following pages.

**Reviewer #1:**

1. The list of references used needs to be updated. Of the 32 papers used, 24 (75%) were published earlier than 2019 (more than 5 years ago), including 5 papers published in 2004 and earlier (20 or more years ago).
The text contains a number of typographical errors (e.g., on line 82) as well as some syntylic errors. I recommend performing English proofreading by a native speaker.
In general, the paper is technically sound, and the methods are appropriate and properly conducted. The results and conclusions logically follow from the conducted analysis. The data are adequately interpreted. The statistical analysis is sound. The claims are fully supported by the study data. There are no special ethical concerns regarding the use of human subjects. I have no other special comments on the work, and I recommend it for publication after minor revision.
Thank you for submitting your study to the Journal and good luck for the paper.

Some of the references have been updated with relevant, which showed in the revised manuscript.

**Reviewer #3:**

Still you didn't answer my question about Figure 1. Figure 1 is study flow diagram, but you mentioned Figure 1 is KM plot of excluded cases in the "Author's Response to Reviewer's Comments". I still need to see the outcome of excluded cases. Same as Figure 2 which you mentioned "KM plot of aggressive group" which I don't see...

Both of the figures were attached in “ Response to Reviewers” instead of manuscript. We will redefined them as Figure 3 and Figure 4.

Figure 3: KM Plot of excluded cases (Marfan, Maximum aortic diameter > 45mm patient) vs. included cases

Excluded cases

Included cases

p = 0.002

Figure 4: KM Plot of aggressive group (Excluded cases vs included cases)

From Figure 3 and 4, patient with Marfan syndrome and maximum aortic diameter > 45mm had worse survival outcome than included cases, with statistically significant. This indirectly supports the hypothesis that the maximum aortic diameter is a significant factor influencing early mortality.

There were 3 cases (7.9%) with Marfan or maximum aortic diameter >45mm in the conservative group. On the contrary, there were 18 cases (18.4%) with Marfan or maximum aortic diameter >45mm in the aggressive group. Hence, if we did not exclude these patients, the power of comparison may be not enough. Furthermore, most patients whose maximum aortic diameter >45mm received pre-emptive TEVAR. In consideration of these factors, we excluded these cases in our study at last. The above Figure 4 was added in the discussion section of revised manuscript (showed as Figure S1).
